# Role of MRI for assessment of GI bleeding: a pictorial review of indications, technique and performance

**DOI:** 10.1007/s00261-025-05065-w

**Published:** 2025-06-28

**Authors:** Haresh V. Naringrekar, Avneesh Gupta, Jeff L. Fidler, Bari Dane, Alexis M. Cahalane, Mike L. Wells

**Affiliations:** 1 Thomas Jefferson University Hospital, Philadelphia, USA; 2 Boston University, Boston, USA; 3 Mayo Clinic, Jacksonville, USA; 4 New York University, New York, USA; 5 Massachusetts General Hospital, Boston, USA

**Keywords:** GI Bleeding, MR Enterography, MRI, CT Enterography, Small Bowel Masses, Small BowelVascular Malformations

## Abstract

The evaluation of patients with gastrointestinal (GI) bleeding is complicated due to the variety of tests available and the large number of potential causes of bleeding. MRI is less commonly used than computed tomography and endoscopy but it can diagnose disease that causes GI bleeding and serve as a complementary role to other tests. MRI is most often used in the form of magnetic resonance enterography (MRE) to assess patients with suspected bleeding from the small bowel. While CT enterography (CTE) and video capsule endoscopy (VCE) are the more commonly used tests in the setting of GI Bleeding, MRE has characteristics which may make it the more favorable modality for a given patient. Potential advantages of MRE, protocol considerations and the literature delineating its diagnostic performance for detecting pathology which can cause GI bleeding relative to CTE and VCE are reviewed here. MRI is uncommonly used to assess patients with upper GI bleeding, lower GI bleeding and patients with bleeding sites that remain undetected despite a formal evaluation of the GI tract, however it may add value in specific clinical scenarios. These uncommon scenarios and specific clinical examples are also presented to highlight the potential benefits of MRI.

## Introduction

**Fig. 1:**
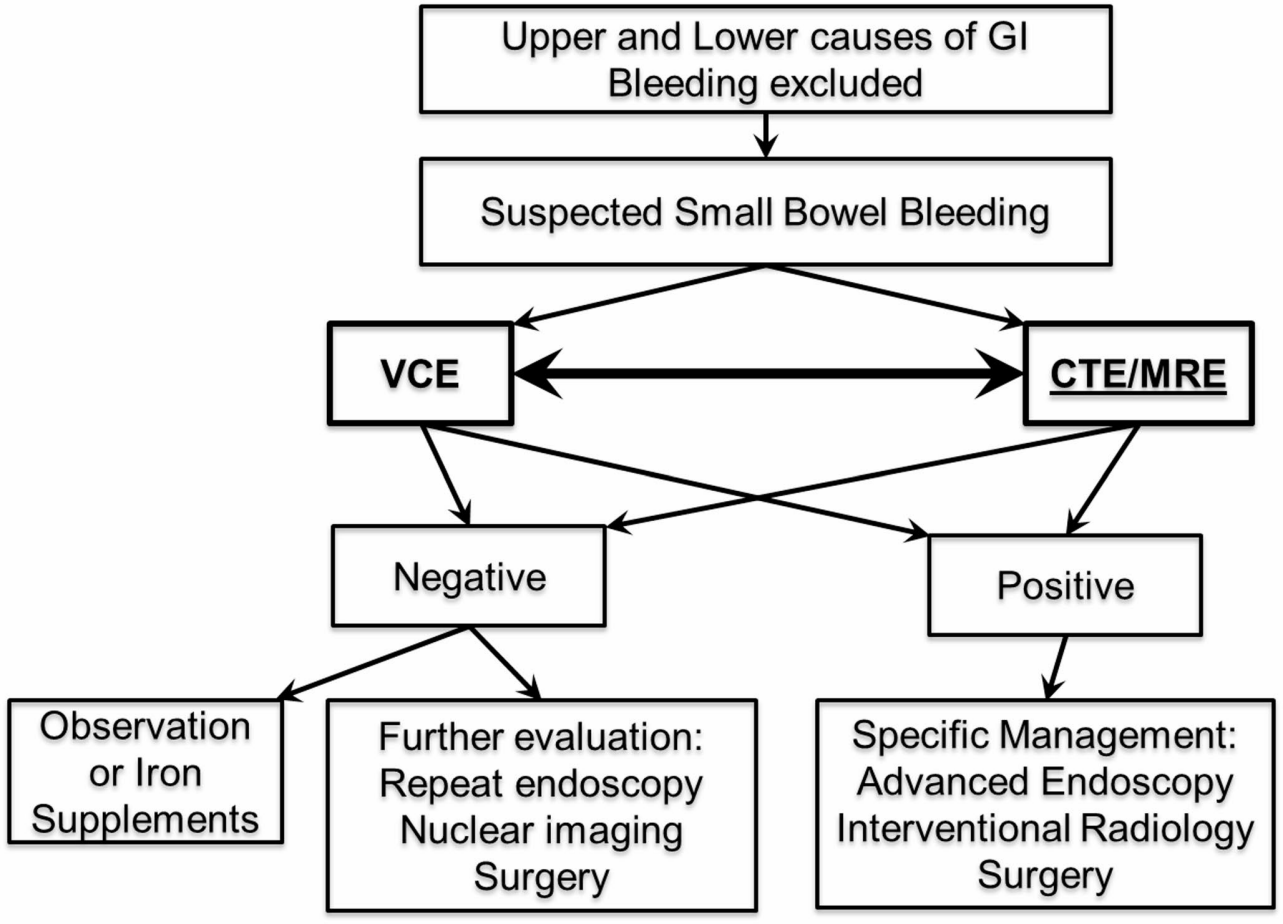
Diagnostic algorithm in the evaluation of patients with suspected small bowel bleeding

Gastrointestinal (GI) bleeding is a major healthcare problem in the United States, being the most common GI diagnosis leading to hospitalization, and leading to millions of annual office visits and several billion dollars in annual health care cost [1]. There are numerous potential sources of GI bleeding and correct diagnosis often requires evaluation with a combination of endoscopic and radiologic tests. Upper GI (UGI) tract bleeding, defined as bleeding occurring in the esophagus, stomach and duodenum up to the Ligament of Treitz, is the most common source of GI bleeding accounting for 80% of cases [2, 3]. Lower GI (LGI) tract bleeding, which occurs distal to the Ligament of Treitz, is the second most common cause of GI tract bleeding accounting for 15–30% of cases [2, 3]. The lower GI tract is further subdivided into the small bowel (SB) and the colon. In the outpatient setting UGI and LGI bleeding are typically first evaluated with esophagogastroduodenoscopy (EGD) and colonoscopy (Fig. 1). If EGD and colonoscopy have failed to identify a source of bleeding, a patient then has “suspected SB bleeding” which accounts for 5–10% cases of GI bleeding [4]. Diagnosing the etiology of SB bleeding is particularly challenging and often requires both advanced endoscopic techniques such as video capsule endoscopy (VCE) and imaging with either computed tomography enterography (CTE) or magnetic resonance enterography (MRE) [3, 4]. If no source of bleeding has been identified despite evaluation of the entire GI tract, a patient then has “obscure GI bleeding”. The subsequent steps in evaluation and management of a patient with obscure GI bleeding depend on many clinical factors, but may involve observation, repeating diagnostic tests or performing less common tests such as device assisted endoscopy or nuclear imaging.

MRI is most commonly used for evaluation of suspected SB bleeding in the form of MRE and can detect a variety of SB pathology which may result in bleeding and will be the focus of review in this article. MRI may also be beneficial for patient evaluation in the setting of UGI and LGI bleeding and for assessing obscure GI bleeding. Use of MRI in these settings is less common, however, clinical scenarios in which MRI may be appropriate for these indications and clinical examples will also be reviewed.

## MR enterography for assessment of suspected small bowel bleeding

### MRE protocol

#### Patient preparation

MR enterography (MRE) requires bowel preparation to optimize visualization of the bowel. Patients typically fast for 4–6  hours prior to the examination, which minimizes filling defects within the SB and improves oral contrast consumption adherence. The target volume for oral contrast varies between institutions, but typical volumes range between 1000 and 2000 mL, with ingestion occurring at a consistent rate over at least 45–60 min to achieve uniform SB distention [5]. Commonly utilized oral agents include commercial flavored sugar beverages (Breeza, Beekly Medical and CitraClear, Bracco Diagnostics inc.), mannitol solution, and polyethylene glycol. Image acquisition is usually performed 45–70 min after oral contrast ingestion. Both the volume of contrast ingestion and time of oral contrast injections are decreased if the patient has surgically altered anatomy such as a colectomy or short bowel. Many imaging centers utilize pharmacologic bowel paralytic agents such as glucagon to improve image quality. In general, these agents are recommended to decrease artifacts on motion-sensitive sequences such as 3D gradient echo (GRE) acquisitions, however the time and expense needed to administer these agents may not be practical for all radiology practices [5].

#### Pulse sequences

An example MRE protocol for assessment of GI bleeding and examples of pathology to be identified are provided in Table 1. Pulse sequences included in a MRE protocol vary in type and acquisition plane between institutions [5]. In general the coronal imaging plane is favored for visualizing the small bowel and can limit the number of image slices needed for coverage. Performing additional sequences in the axial plane can be helpful as certain abnormalities may be more conspicuous, and there may be less artifact (particularly for diffusion-weighted imaging (DWI)); in the uncommon event of active bleeding axial images may better demonstrate extravasated contrast material pooling along the dependent side of the bowel wall. Commonly included sequences include T2 weighted single shot fast spin echo (T2 SSFSE) with and without fat-suppression, steady state free precession (SSFP), DWI, and T1 weighted sequences. Fat-suppressed T1 weighted pre-contrast and post-contrast images are typically acquired at high resolution with 3-D GRE in the coronal plane followed by axial acquisition. Pre-contrast imaging is necessary to identify high T1 signal intensity in the bowel which may obscure enhancing pathology or active bleeding. During imaging, a phased array surface coil is used to cover as much bowel as possible. Imaging in the prone position has the advantages of decreasing abdominal wall motion artifacts, flattening the abdominal wall which decreases coronal acquisition slice number and scan time, and may speed gastric emptying.

**Table 1:** Example MRE protocol sequences and specific pathologies best seen on each acquisition

Sequence	Coverage	TR/TE	Slice (mm)	FOV (mm)	Notes
Coronal T2 SSFSE	Abdomen, Pelvis	900/90	6	372 × 452	Evaluation of bowel anatomy, wall thickness, fold pattern and caliber. Can depict post-surgical changes in the bowel or anatomic variants such as Meckel’s diverticulum
Coronal T2-FS SSFSE	Abdomen, Pelvis	900/90	6	372 × 452	Improved visualization of bowel wall edema and inflammation
Axial T2 SSFSE	Abdomen, Pelvis	900/90	6	370 × 370	Evaluation of bowel anatomy
Axial DWI (b = 0, 800)	Abdomen, Pelvis	7600/54	6	340 × 370	Detection of bowel inflammation and masses
Coronal T1-FS pre- and post dynamic contrast enhanced series	Abdomen, Pelvis	4.08/1.65	3	325 × 325	Post contrast dynamic phases typically include arterial and portal venous phases. Used for delineation of bowel vascular anatomy, and detection of enhancing bowel masses, inflammation, vascular malformations and active bleeding
Axial T1-FS post-contrast	Abdomen, pelvis	3.07/1.47	2.5- 3	325 × 325	
Coronal T1-FS post-contrast	Abdomen, Pelvis	4.56/2.38	3	256 × 179	Detection of delayed phase hyperenhancing stricture or mass. Can be helpful for showing luminal contrast accumulation in patient with active bleeding

### Strengths and limitations of MRE relative to CTE

**Fig. 2:**
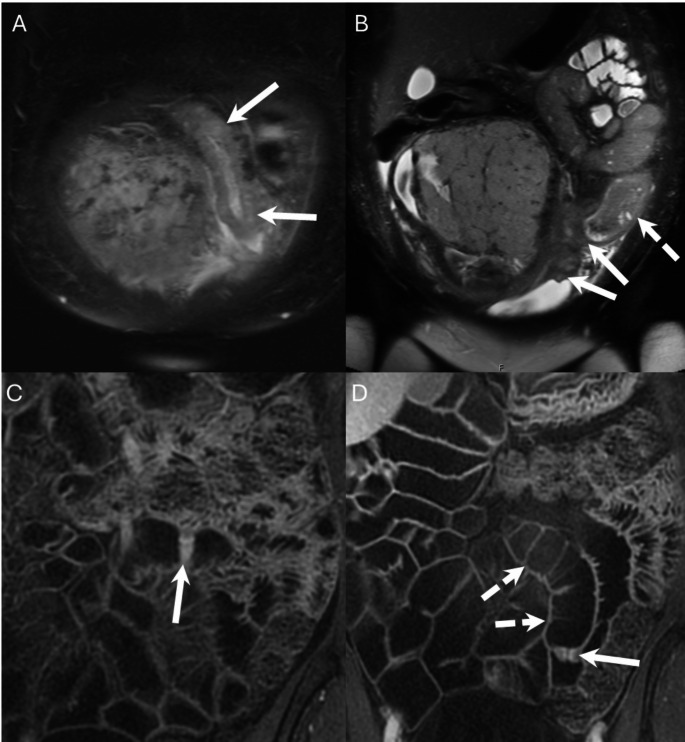
**A**,** B** 23-year-old female pregnant at 22 weeks 1 day. Patient presents with progressively worsening generalized abdominal pain for 2 months, anemia and rectal bleeding previously ascribed to hemorrhoids. Non contrast MRI was obtained primarily to assess for causes of abdominal pain. (**A**,** B**) Coronal T2 fat saturated images show marked thickening and edema of a long segment of the distal ileum (arrows, **A**), a fistula between the ileum and urinary bladder (arrows, **B**) and pre-stenotic bowel dilation (dashed arrow, **B**). **C**, **D** 64-year-old female with chronic low back pain and anemia treated with iron supplementation. Coronal post gadolinium images demonstrate several diaphragm type strictures, two of which are shown (arrows, **C** and **D**), associated with mild dilation of the upstream bowel (dashed arrows, **D**). The findings are consistent with diaphragm disease and the patient was found to be taking high doses of NSAID medications

**Fig. 3:**
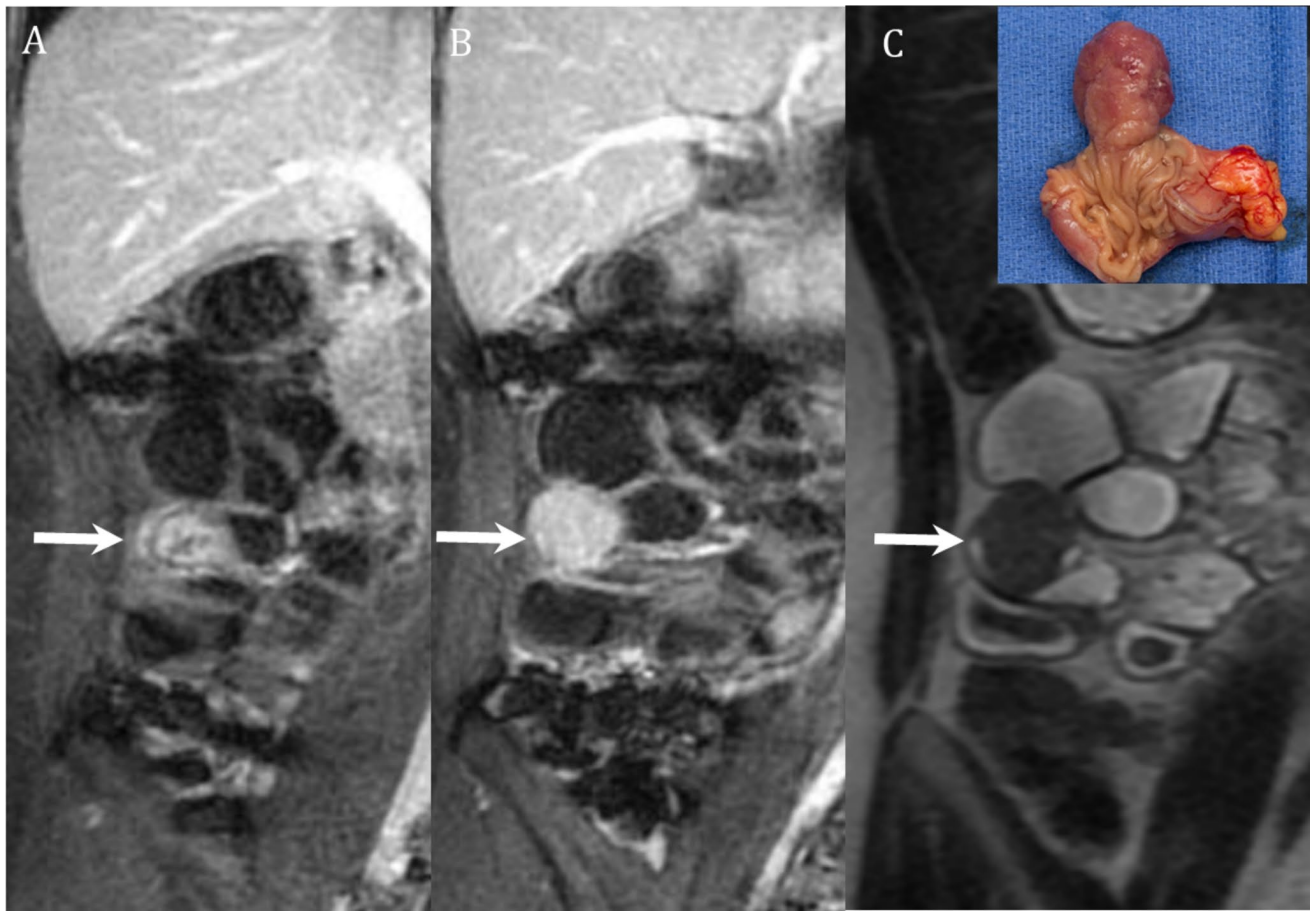
28-year-old female with intermittent abdominal pain and chronic anemia. MRE with coronal postcontrast T1 images in the arterial (arrow, **A**) and delayed (arrow, **B**) phases demonstrates a progressively enhancing mass arising from ileum with low signal on coronal T2 SSFSE (arrow, **C**). Subsequent surgical resection demonstrated a hamartomatous polyp (inset image, **C**)

**Fig. 4:**
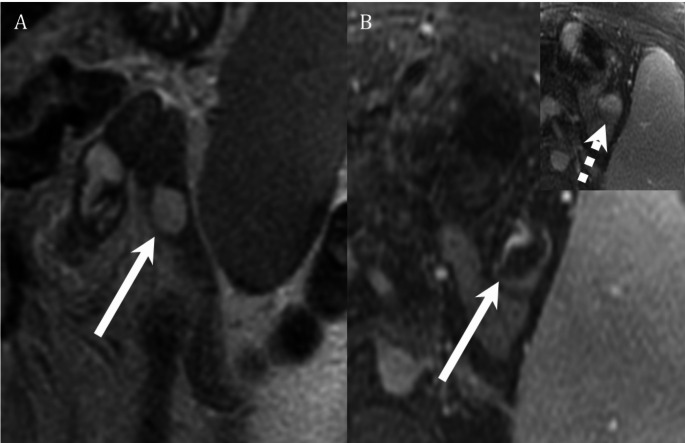
70-year-old female being evaluated with abdominal MRI for cirrhosis. **A** Coronal T2 SSFSE demonstrates a mass within the jejunum with high T2 signal, similar to that of luminal fluid (arrow, **A**). **B** Axial post gadolinium T1FS images in the portal venous and delayed phases (inset image, **B**) demonstrate slow, progressive peripheral enhancement (white arrow, **B**) with complete fill in on the delayed phase (dotted arrow, inset image). The finding was suspected to represent a hemangioma or slow flow vascular malformation and was unchanged on 1 year follow up MRI (not shown)

CTE is often preferred over MRE for the evaluation of SB bleeding primarily due to its wider availability and ease of use, however, MRE offers several potential benefits. A major advantage of MR is the lack of ionizing radiation, which is particularly important when assessing pediatric patients, young adult patients, or pregnant patients wishing to avoid fetal radiation exposure [6] (Fig. 2). MRI obtains images at several time points and with multiple phases of contrast enhancement without the additive radiation dose which is associated with CT scanning. Obtaining imaging at multiple time points allows for each segment of the bowel to be assessed in good distention; this is particularly beneficial for assessment of the jejunum which can be difficult to adequately distend. Optimal bowel distention is important for detection of pathology that may be a source of GI bleeding, and it increases confidence that a finding represents a true lesion and not under distended bowel (Fig. 2). Obtaining multiple phases of contrast enhancement on each case is important for several reasons, including detection of masses which may have maximal contrast compared to background bowel at either an early or late phase of enhancement and for delineating the pattern of enhancement over time which is important for defining the type of mass being observed (Figs. 3 and 4). Acquiring multiple contrast enhanced phases may also allow improved detection of active bleeding by better demonstrating accumulation of contrast material in the bowel lumen over time.

**Fig. 5:**
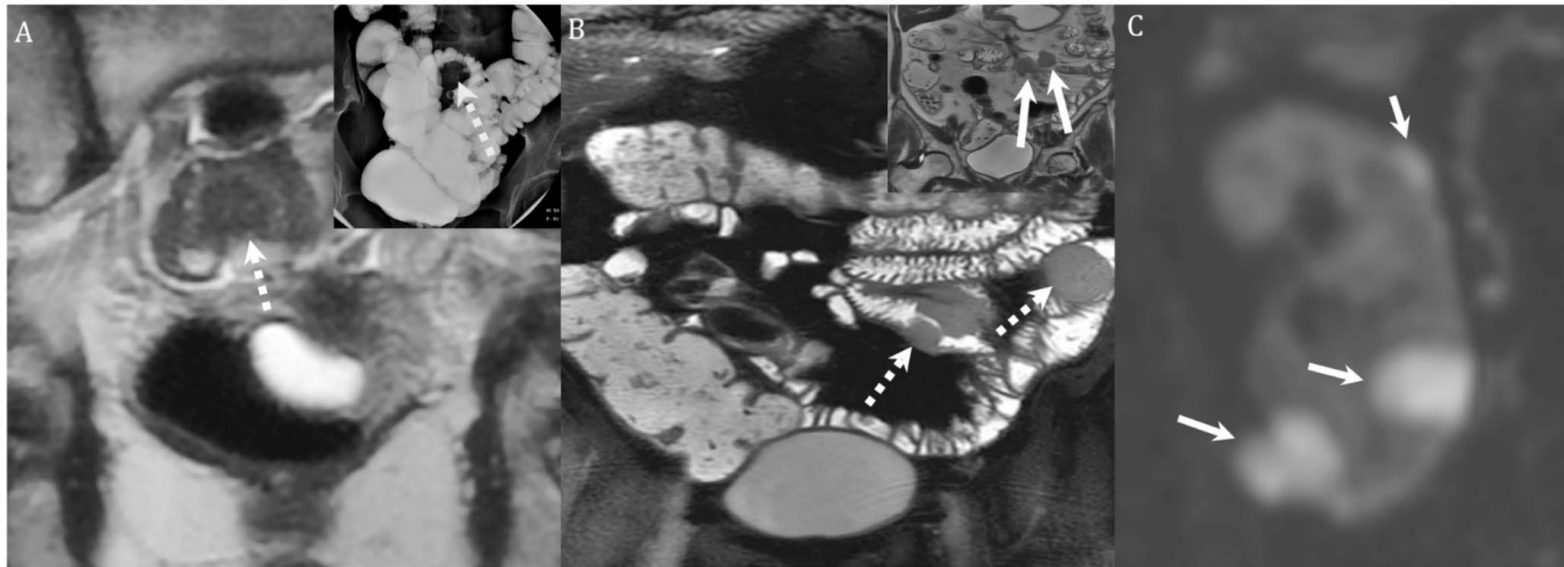
**A** 52-year-old female underwent routine colonoscopy with inability to advance scope beyond an angulated sigmoid stricture. Barium enema demonstrates a short segment colonic stricture (arrow inset image, **A**). Coronal T2 image from MRE shows this stricture to be caused by a T2 hypointense endometriotic implant with mushroom cap appearance (dotted arrow, **A**). **B**,**C** 62-year-old patient with anemia and suspected small bowel bleeding was found on MRE to have findings of lymphoma including multiple large but non-obstructing masses in the small bowel (dotted arrows, **B**) and bulky mesenteric lymphadenopathy (arrows inset image, **B**) on coronal T2 weighted sequences. Coronal diffusion weighted imaging from the exam also showed multiple masses within the kidneys (arrows, **C**)

IV gadolinium contrast agents have a lower incidence of allergic or adverse reaction than iodinated contrast agents [7]. Patients with an allergy to iodinated contrast media or renal impairment may be better served with a gadolinium enhanced MRE [8]. Additionally, patients who cannot receive IV gadolinium (pregnant patients) or in patients who may have difficulty completing a full MRE protocol such as the pediatric population may benefit from an abbreviated unenhanced MRE to evaluate for sources of bleeding. (Figs. 2 and 5) An abbreviated non-IV contrast enhanced MRE protocol including SSFSE without and with fat saturation, SSFP and DWI sequences would be capable of detecting pathology such as mass, stricture or inflammation [9–11]. These sequences and T1 weighted images maybe able to identify hematoma in the bowel of a patient with active or recent bleeding..

The multiphasic postcontrast imaging and high tissue contrast images obtained at MRE can facilitate assessment of extraintestinal abnormalities. This includes lesions such as endometriosis or malignancies which may be difficult to characterize on CTE. MRE’s ability to assess extraintestinal structures to facilitate staging of malignancies involving the bowel may have implications for the patient’s goals of care regarding management of their bleeding [12] (Fig. 5).

There are several disadvantages of MRE compared to CT including more limited access, length of the exam which may lead to decreased patient cooperation and motion artifacts, more variability in exam quality and required expertise for interpretation. MRE is generally not indicated for patients who need urgent evaluation or who may become hemodynamically unstable due to limited immediate availability, the need to screen and identify metallic implants prior to imaging and long acquisition times. In these cases, obtaining a CT is more practical and is the favored imaging modality. MRE has a higher potential for image artifacts and has relatively lower spatial resolution when compared to CTE. Both factors can impair detection of small or subtle abnormalities such as vascular malformations. For these reasons, the American College of Radiology appropriateness criteria states that MRI is usually not appropriate for active or ongoing LGI bleeding but may be appropriate in stable patients with obscure recurrent LGI bleeding or as an initial imaging study for non-variceal upper GI bleeding after a negative endoscopy [13, 14]. The use of MRI for detection of gastrointestinal bleeding is therefore mainly focused on occult suspected SB bleeding.

### Performance of MRE relative to CTE and VCE for detection of small bowel disease known to cause GI bleeding

Patients with suspected SB bleeding are most commonly evaluated with video capsule endoscopy or CTE to determine the underlying cause of the bleeding. There is scarce data available for direct comparison between MRE and these modalities in patients presenting with GI bleeding, or for detection specifically of active bleeding. However, the primary goal of enterography is to identify underlying pathology that may be the source of bleeding such as inflammation or a mass. There are several publications available directly comparing the ability of MRE to detect small bowel pathology, against that of VCE and CTE. For example, a study comparing CT enteroclysis with a limited MR enteroclysis protocol in 50 patients referred for a variety of suspected SB disease found that MR had both lower sensitivity and lower interobserver agreement [15]. A more recent study directly comparing 3 phase CTE to MRE in 150 patients suspected of having a variety of SB disease found MR enterography to be significantly more sensitive and accurate for detection [16].

Most available studies comparing VCE with MRE have found higher sensitivity for identifying pathology at VCE [17–20]. One study comparing MR enteroclysis with VCE in patients with suspected SB disease did find an overall similar test performance between the two modalities, but with better overall specificity for MR enteroclysis [10].

Small bowel mass lesions such as polyps, metastases and primary small bowel tumors are a known common cause of GI bleeding, and MR enterography has been shown in prior studies to perform well for mass detection [2]. Prior direct comparison with CTE and MRE has shown better detection of SB masses with MRE, particularly for masses within the jejunum which is often difficult to adequately distend at enterography [16]. Additional studies have shown very good performance for mass detection with MR enteroclysis (Sensitivity (Se) 91–94%, Specificity (Sp) 95–97%, interobserver agreement (K) = 0.095) and MRE (Se 86%, Sp 98%) [21, 22]. In a study of patients with polyposis, MRE performed well for detection of polyps > 15 mm in size and had better sensitivity when used with IV contrast [11]. Several studies comparing MRE with VCE have shown good performance for mass detection with MRE, demonstrating it has a complimentary role to endoscopic techniques [10, 18, 20]. Some studies suggest that VCE may have an advantage for finding sub-centimeter masses that can be missed due to the relatively low spatial resolution or image artifacts often found at MRE [11, 22].

Inflammatory pathology in the small bowel such as Crohn’s disease, inflammatory strictures or peptic ulcer disease are well known causes of bleeding [2]. Detection of SB inflammation by MRE has best been studied in patients with Crohn’s disease, where it has demonstrated similar sensitivity as CTE (90.5% vs. 95.2%) [16, 23, 24]. MRE can also identify bowel inflammation caused by other etiologies, although its accuracy relative to CT has not been well documented in the literature. MRE has a potential advantage over CTE in detecting strictures caused by inflammation (such as those associated Crohn’s disease or non-steroidal anti-inflammatory drug enteropathy), by providing assessment at multiple times points and at multiple phases of enhancement (Fig. 2) [25]. When compared with VCE, MRE has been shown by meta-analysis to have similar diagnostic yield for detecting active SB Crohn’s disease [26]. Other studies examining VCE and MRE have shown both to be effective at detecting inflammation, and serving as complimentary modalities with each test being able to detect regions of inflammation missed by the other [17, 19].

**Fig. 6:**
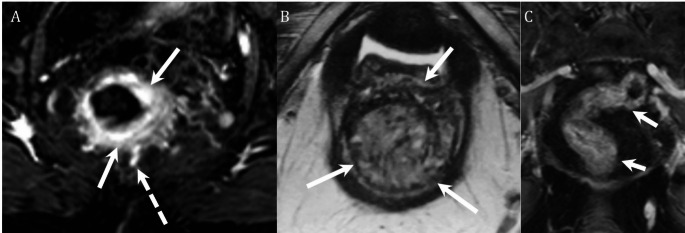
Two different patients presenting with rectal bleeding with etiology diagnosed on MRI. **A** 66-year-old with metastatic renal cell carcinoma developed new rectal bleeding after initiating therapy with pembrolizumab and axitinib. MR was ordered primarily to assess for new metastatic disease. Axial enhanced T1 image demonstrates findings of proctitis including mural thickening and hyperenhancement (arrows, **A**) and engorged vasa recta (dashed arrow, **A**). **B**, **C** 74-year-old female with chronic intermittent vaginal and rectal bleeding. Colonoscopy 10-years previously was normal. Axial T2 image from pelvic MRI demonstrates a large T2 hyperintense mass involving the rectum, anal canal, and posterior vaginal wall (arrows, **B**). Coronal enhanced T1 image shows an irregular pattern of enhancement diffusely throughout the rectal portion of the mass (arrows, **C**). The imaging findings were most consistent with a hemangioma and the mass was followed with imaging showing slow growth over the course of several years

Small bowel vascular malformations are among the most common causes of GI bleeding, particularly for adult patients [2]. MRE has consistently been shown to perform poorly for detecting small vascular malformations such as angioectasia when compared with endoscopic methods such as VCE [10, 17–20]. MRE has also shown worse performance for detection of vascular malformations relative to CTE [16]. These findings are not unexpected given the lower spatial resolution and artifacts on MRE compared to CTE. While MRE may be able to identify and assess larger vascular malformations (Figs. 4 and 6), it should not be considered an effective modality to identify small or flat vascular malformations. Exam characteristics for MRE vs. CTE and VCE are summarized in Table 2.

**Table 2:** Summary table comparing MRE, CTE and VCE characteristics for evaluation of patients with suspected small bowel bleeding

	Small bowel mass detection	Small bowel inflammation detection	Vascular malformation detection	Radiation exposure	Image resolution	Contraindications	Patient population
MRE	+++	+++	+	n/a	+	Incompatible implant or prosthetic Inability to lie flat for extended period of time Rapid bleeding or Hemodynamic instability	Patients wishing to avoid radiation exposure (children, pregnancy) Patients who require imaging and cannot receive iodinated contrast
CTE	+++	+++	++	++	++	Severe iodinated contrast allergy	First line imaging test for most patients
VCE	++	+++	+++	n/a	+++	Clinical concern for stricture or bowel obstruction Pregnancy	Considered first line test along with CTE for most patients

### Use of MRI for evaluation of upper and lower GI bleeding

**Fig. 7:**
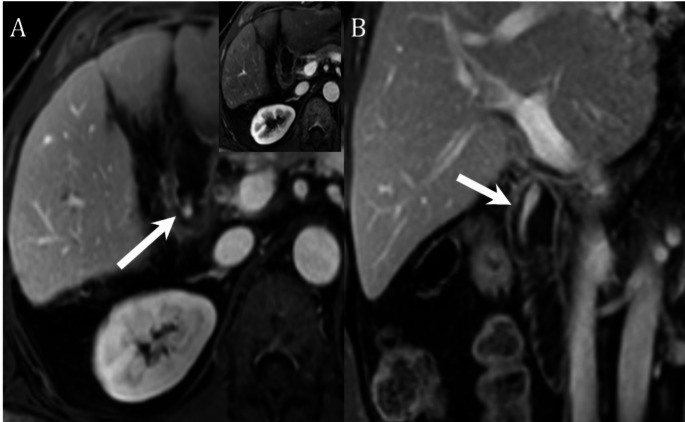
60-year-old male liver transplant patient with clinical suspicion of upper GI bleeding and mild elevation of liver function tests. MRCP performed for assess the liver and postoperative anatomy prior to endoscopy. When compared with arterial phase (inset image **A**), there is new active bleeding in the descending duodenum on portal venous (arrow, **A**), and coronal 90 s delayed (arrow, **B**) images

Upper gastrointestinal sources of bleeding are typically assessed with endoscopy, but in uncommon clinical scenarios with suspected slow or intermittent UGI bleeding, MRI may be performed prior to endoscopic assessment (Fig. 7). When compared with CT, abdominal MRI has an advantage for evaluation of the biliary tree, detection and characterization of liver masses and evaluation of pancreatic pathology which can lead to UGI bleeding. Multiphasic post-gadolinium MRI imaging can also demonstrate active bleeding similar to a CTA exam (Fig. 7) [27]. Clinical scenarios might include stable bleeding patients with hepatobiliary or pancreatic malignancy in which evaluation of the extent of disease is needed before performing endoscopy, or patients who have undergone an interventional procedure potentially resulting in hemobilia in which the post-procedural anatomy warrants assessment. In patients with suspected hemobilia or hemosuccus pancreatitis, MRCP may be valuable for assessing the pancreatic parenchymal and ductal anatomy before planning an intervention to treat the bleeding.

Typically, LGI bleeding and anorectal pathology are best assessed with colonoscopy. Like the UGI tract, MRI may uncommonly be used to assess for anorectal pathology in bleeding patients with complicated or unclear clinical evaluation. MRI is routinely used to stage anorectal carcinomas and further delineate extent of other neoplasms found on endoscopy. MRI can also be helpful for assessing the location and extent of vascular malformations or inflammatory processes involving the rectum and anus [28] (Fig. 6).

## MRI assessment of active bleeding in obscure GI bleeding

**Fig. 8:**
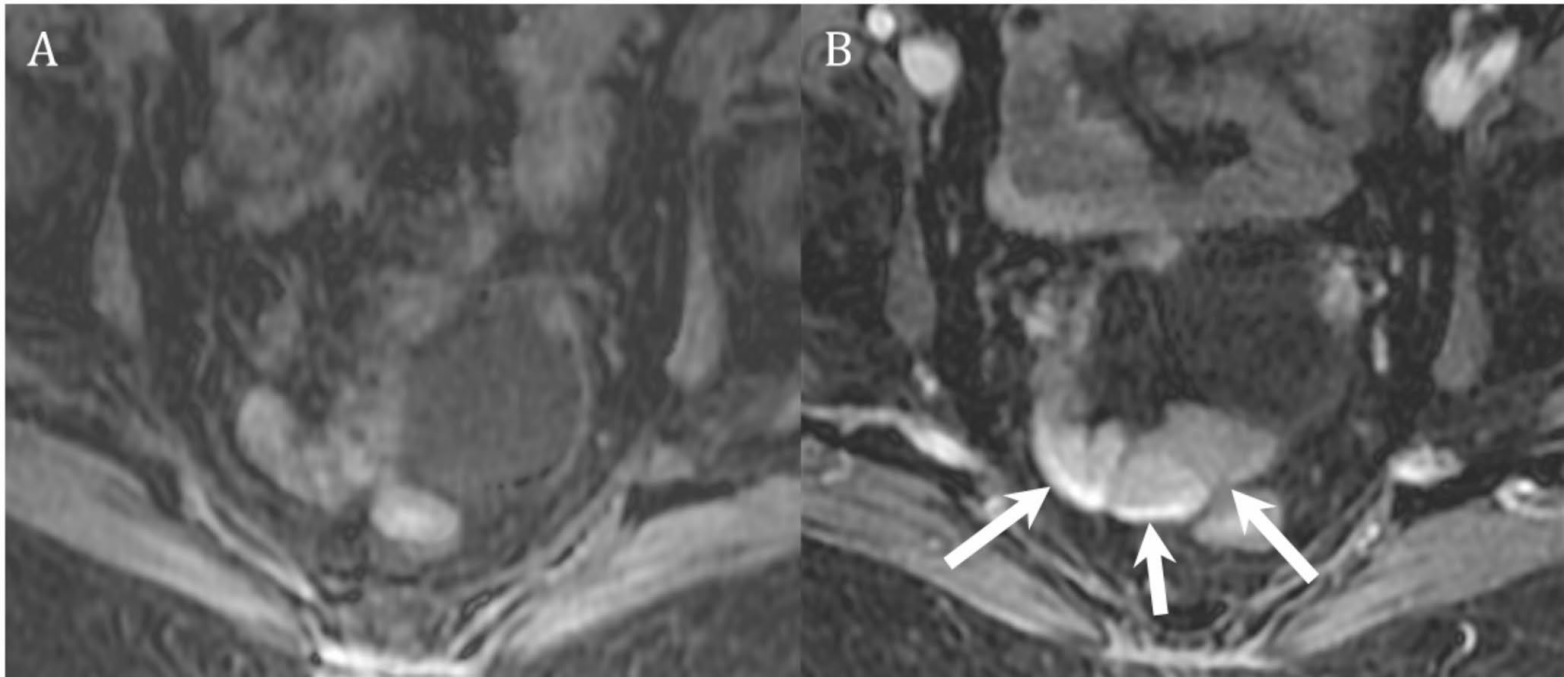
79 year old female presenting with recurrent overt obscure GI bleeding. Prior workup included upper and lower endoscopy, and VCE which demonstrated bleeding in the mid-small bowel. Attempted retrograde double balloon endoscopy failed due to tortuosity of the bowel. Axial precontrast (**A**), 30 min delayed (not shown) and 3 h delayed (**B**) postcontrast imaging from MRI enhanced with ferumoxytol demonstrates accumulation of contrast in the distal ileum (arrows, **B**). The patient underwent targeted fluoroscopic angiography which identified and embolized an ileal angioectasia

Gadolinium enhanced MRI can demonstrate active extravasation in the bowel lumen (Fig. 7). Using contrast agents with long blood pool circulation times, MR can have the additional benefit of demonstrating slow active extravasation over a prolonged period. Ferumoxytol enhanced MRI, for example, is a novel method for assessment of active bleeding in patients in whom a bleeding source has not been identified after typical imaging and endoscopic methods. Ferumoxytol is an iron based IV contrast agent with a plasma half-life of 14–15 h. The contrast agent allows delayed imaging to be obtained over several days to assess for slow extravasation into the GI tract (Fig. 8). Angiographic MRI and conventional MRE imaging sequences can also be obtained for high quality delineation of the vascular and bowel anatomy to accurately report the location of bleeding, and to search for its underlying cause [27, 29].

## Conclusion

MRI is most commonly used to evaluate GI bleeding patients in the form of MRE, and in the setting of suspected SB bleeding. Both MRE and CTE are considered complementary tests to VCE for detecting small bowel pathology which may cause GI bleeding. MRE performs well for identification of common causes of small bowel bleeding including mass lesions and inflammation, while it performs poorly for identification of vascular malformations. These characteristics are complementary to those of VCE, which excels at identification of vascular malformations and can overlook submucosal mass lesions and inflammation. MRE may be a more appropriate choice for small bowel evaluation than CTE in specific patients. In young and pregnant patients MRE may be preferred due to the lack of ionizing radiation. In patients who cannot receive or refuse IV contrast, a non-contrast MRE can still detect small bowel pathology and may be preferred over CTE. MRE may also be preferred over CTE in patients with clinical suspicion of a small bowel mass, such as a patient with known malignancy with new GI bleeding, or in a patient with high suspicion of bowel inflammation. Although less common, MRI can also be advantageous for assessing UGI and LGI bleeding in specific clinical scenarios, such as delineating anatomy in patients with haemobilia prior to endoscopy, or for assessing anorectal pathology. Novel applications of MRI enhanced with blood pool agents may be helpful for detecting the location of obscure slow or intermittent GI bleeding. The workup of patients with GI bleeding is complex and often requires evaluation with multiple endoscopic and radiologic tests. When used appropriately, MRI serves a complementary role in the evaluation of patients with GI bleeding.

## Data Availability

No datasets were generated or analysed during the current study.
